# IL-21 Expands HIV-1-Specific CD8^+^ T Memory Stem Cells to Suppress HIV-1 Replication In Vitro

**DOI:** 10.1155/2019/1801560

**Published:** 2019-04-30

**Authors:** Kang Wu, Shaoying Zhang, Xu Zhang, Xinghua Li, Zhongsi Hong, Fei Yu, Bingfeng Liu, Ting Pan, Zhaofeng Huang, Xiao-ping Tang, Weiping Cai, Jinyu Xia, Xuefeng Li, Hui Zhang, Yiwen Zhang, Linghua Li

**Affiliations:** ^1^Institute of Human Virology, China; ^2^Key Laboratory of Tropical Disease Control of Ministry of Education, China; ^3^Guangdong Engineering Research Center for Antimicrobial Agent and Immunotechnology, Zhongshan School of Medicine, Sun Yat-sen University, Guangzhou, 510080, China; ^4^National-Local Joint Engineering Research Center of Biodiagnosis & Biotherapy, The Second Affiliated Hospital, Xi'an Jiaotong University, Xi'an, 710004, China; ^5^Department of Infectious Diseases, Fifth Affiliated Hospital, Sun Yat-sen University, Zhuhai, Guangdong, 519000, China; ^6^Department of Infectious Diseases, Guangzhou Eighth People's Hospital, Guangzhou Medical University, Guangzhou, 510060, China

## Abstract

Due to the existence of viral reservoirs, the rebound of human immunodeficiency virus type 1 (HIV-1) viremia can occur within weeks after discontinuing combined antiretroviral therapy. Immunotherapy could potentially be applied to eradicate reactivated HIV-1 in latently infected CD4^+^ T lymphocytes. Although the existence of HIV-1-specific CD8^+^ T memory stem cells (T_SCM_s) is well established, there are currently no reports regarding methods using CD8^+^ T_SCM_s to treat HIV-1 infection. In this study, we quantified peripheral blood antigen-specific CD8^+^ T_SCM_s and then expanded HIV-1-specific T_SCM_s that targeted optimal antigen epitopes (SL9, IL9, and TL9) in the presence of interleukin- (IL-) 21 or IL-15. The suppressive capacity of the expanded CD8^+^ T_SCM_s on HIV-1 production was measured by assessing cell-associated viral RNA and performing viral outgrowth assays. We found that the number of unmutated TL9-specific CD8^+^ T_SCM_s positively correlated with the abundance of CD4^+^ T cells and that the expression of IFN-*γ* was higher in TL9-specific CD8^+^ T_SCM_s than that in non-TL9-specific CD8^+^ T_SCM_s. Moreover, the antiviral capacities of IL-21-stimulated CD8^+^ T_SCM_s exceeded those of conventional CD8^+^ memory T cells and IL-15-stimulated CD8^+^ T_SCM_s. Thus, we demonstrated that IL-21 could efficiently expand HIV-1-specific CD8^+^ T_SCM_s to suppress HIV-1 replication. Our study provides new insight into the function of IL-21 in the *in vitro* suppression of HIV-1 replication.

## 1. Introduction

HIV-1 infection is a chronic disease that suppresses and destroys the immune system [1]. After suppressive combined antiretroviral therapy (cART), associated viremia becomes undetectable. However, the virus can persist in stable reservoirs, primarily in resting memory CD4^+^ T cells, which prevents complete elimination and results in viremia rebound upon cART discontinuation [2–4]. Some small compounds have been developed to reactivate HIV-1 latently infected CD4^+^ T lymphocytes (a process known as “shock”). However, it is also important to develop methods to eradicate these reactivated cells, which express viral antigens (a process known as “kill”). The adoptive transfer of immune cells might be an appropriate method. Some treatments utilize immunotherapies that are not virus-specific to target HIV-1 (e.g., the administration of interferon gamma (IFN-*γ*) or the transfer of autologous dendritic cells or nonspecific cytotoxic T lymphocytes (CTLs)). Nevertheless, none of these clinical practices have achieved satisfactory efficacy [5, 6]. CTLs play a pivotal role in the suppression of HIV-1 replication. However, due to selective pressure, the virus can evade CTL recognition by acquiring mutations. The study of CTL escape has led to the development of CTL-based vaccines that exploit conserved viral epitopes (such as HLA-A2-restricted SLYNTVATL (SL9) and HLA-A2-restricted ILKEPVHGV (IL9)). However, targeting these conserved epitopes has not resulted in adequate effects. Therefore, based on recent advances in antigen-specific immunotherapies for tumor suppression, it is reasonable to target nonmutated HIV epitopes and adapt various virus-specific immunotherapies to eradicate reactivated HIV-1 in latently infected CD4^+^ T lymphocytes.

Human CD8^+^ T memory stem cells (T_SCM_s) are a newly identified subset of postmitotic CD8^+^CD45RO^−^CD45RA^+^CD62L^+^CCR7^+^ T cells that express high levels of surface proteins such as CD95 (also called Fas or APO-1) and CD122 (the common interleukin- (IL-) 2 and IL-15 receptor beta chain); they can be efficiently generated via activation of the IL-7 and IL-15 signaling pathways [7–10]. Self-renewing, multipotent CD8^+^ T_SCM_s exist among the peripheral blood mononuclear cells (PBMCs) of healthy individuals [7]. They have also been identified in individuals with Chagas disease and in HIV-1-infected individuals [7, 11, 12]. Although the existence of CD8^+^ T_SCM_s has been established, antigen-specific CD8^+^ T_SCM_s (especially HIV-1 antigen-specific) have not been well characterized. Compared to conventional memory T cells, CD8^+^ T_SCM_s clear pathogens more rapidly and efficiently [7]. Therefore, HIV-1-specific CD8^+^ T_SCM_s might be appropriate candidates for HIV-1 immunotherapy [13].

The common cytokine receptor gamma-chain (encoded by *IL2RG*) is shared by receptors for IL-2, IL-4, IL-7, IL-15, and IL-21. Further, the persistence of T_SCM_s is dependent on the activation of IL-15 signaling [14–16]. The cytokine IL-21 has broad pleiotropic actions that affect the differentiation and function of immune cells such as natural killer cells, B cells, and T cells [17]. IL-21 administration has facilitated the generation of chimeric antigen receptor-expressing T_SCM_s specific for CD19 for the treatment of human B cell malignancies [18]. Furthermore, in CD8^+^ T cells isolated from HIV-1-infected individuals, the expression of perforin is enhanced in response to IL-21 compared with that in response to IL-15 [19]. Moreover, the treatment of cancer or HIV-1 patients with recombinant IL-21 augments immune activities such as the production of IFN-*γ* and perforin [17, 20, 21]. Interestingly, HIV-1-specific CD8^+^ T cells can express IL-21 after primary infection and the IL-21-expressing cell population is expanded in individuals referred to as elite controllers of HIV-1 infection [22]. Thus, we explored optimal approaches to generate HIV-1 antigen-specific CD8^+^ T_SCM_s, via IL-21-mediated expansion, to eradicate reactivated HIV-1 in latently infected cells.

In this study, we identified HIV-1-specific T_SCM_s that targeted conserved epitopes in HIV-1-infected individuals. We found that treatment with IL-21 enhanced the expansion and antiviral function of HIV-1-specific CD8^+^ T_SCM_s. Moreover, when IL-21-generated CD8^+^ T_SCM_s were cocultured with PBMCs from HIV-1-infected individuals, they could further inhibit HIV-1 replication as compared to the conventional memory T cells or IL-15-generated CD8^+^ T_SCM_s. Taken together, we demonstrated that IL-21 expands HIV-1-specific CD8^+^ T_SCM_s, which can efficiently eliminate HIV-1 in latently infected CD4^+^ T cells.

## 2. Materials and Methods

### 2.1. Patient Cohort

Our research was approved by the Ethics Review Board of Guangzhou Eighth People's Hospital (Guangzhou Infectious Disease Hospital, Guangzhou, China) and the Ethics Review Board of Sun Yat-sen University. HIV-1-infected individuals were recruited at Guangzhou Eighth People's Hospital. All study participants provided written informed consent. All patients (who were predominantly human leukocyte antigen- (HLA-) A^+^) were recruited based on the prolonged, continuous suppression (for approximately 1 year) of plasma HIV-1 viremia while on cART, which was defined as results below the detection limits of standard clinical assays (<20 copies of HIV-1 RNA/ml). The patient CD4^+^ T cell counts were <100 cells/mm^3^ (<50 CD4^+^ T cells/mm^3^ in some patients) at the time of initial diagnosis. Anonymized human PBMCs from healthy blood donors were provided by the Guangzhou Blood Center. We confirmed that all methods were performed in accordance with the relevant guidelines and regulations.

### 2.2. Real-Time Quantitative Reverse Transcription Polymerase Chain Reaction

Total RNA was isolated using TRIzol® reagent (Life Technologies, Carlsbad, CA, USA). cDNA was synthesized with the PrimeScript™ RT Reagent Kit (Takara, Shiga, Japan). All primers were annealed at 37°C and reverse transcription (RT) proceeded at 42°C. Quantitative polymerase chain reaction (PCR) was performed with a SYBR® Premix Ex Taq™ II Kit (Takara, Shiga, Japan) according to the manufacturer's instructions. The expression of viral RNA was determined by real-time quantitative RT-PCR (qRT-PCR) with the primer pair SK38 (5′-ATAATCCACCTATCCCAGTAGGAGAAA-3′) and SK39 (5′-TTTGGTCCTTGTCTTATGTCCAGAATGC-3′). For the absolute quantification of cell-associated HIV-1 copies under various treatments, the *in vitro*-synthesized HIV-1 RNA was used as an external control for the measurement of cell-associated viral RNA, as previously described [23–25]. For the relative quantification of cell-associated HIV-1 RNA, the expression of the CD4 gene was determined by real-time quantitative RT-PCR (qRT-PCR) with the primer pair CD4 forward (AAGGACAGCAAGGATCCCCT) and CD4 reverse (GAAGATGAGGGTGCACGAGT) as the internal control.

### 2.3. Peptide-Specific Stimulation

**Figure 1:**
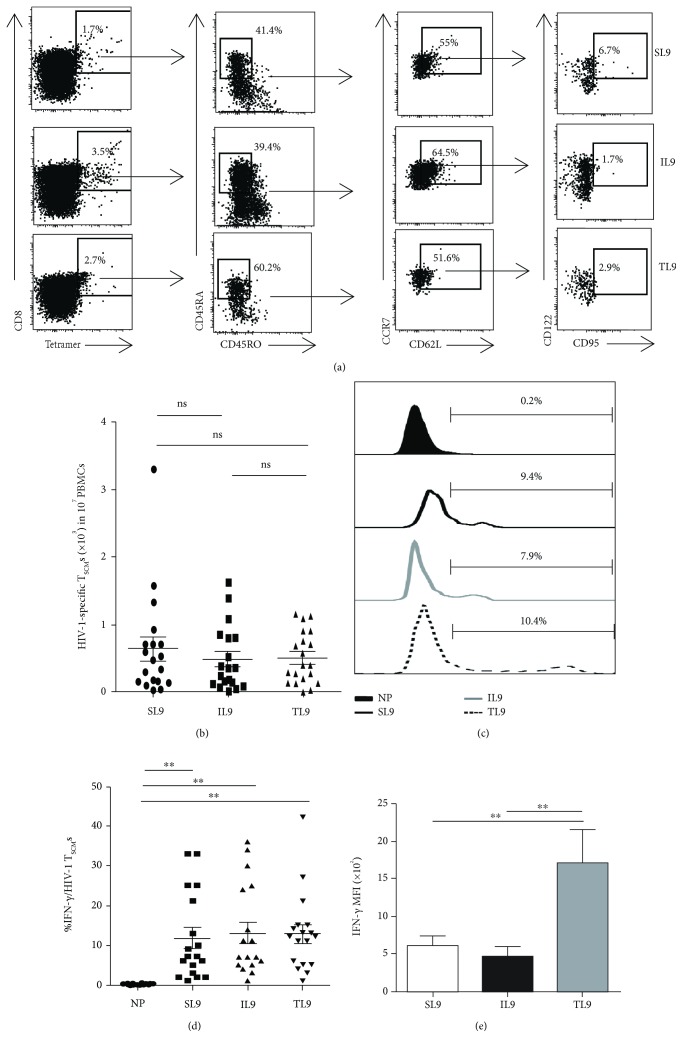
Identification of human immunodeficiency virus type 1- (HIV-1-) specific CD8^+^ T memory stem cells among the peripheral blood mononuclear cells of HIV-1-infected individuals. (a) Flow cytometric analysis of peripheral blood mononuclear cells (PBMCs) from HIV-1-infected individuals. Dot plots show the gating strategy to identify HIV-1-antigen-specific CD95^+^CD122^+^CD8^+^ T memory stem cells (T_SCM_s). The numbers in the graphs show the percentages of subsets. The data are representative of five independent experiments (*n* = 6). (b) The percentages of circulating HIV-1-specific CD8^+^ T_SCM_ subsets in 20 HIV-1-infected individuals are shown as the mean ± standard error of the mean (SEM). A Kruskal–Wallis test with Dunn's multiple comparisons was performed to assess statistical significance. (c) Intracellular interferon gamma (IFN-*γ*) staining in PBMCs from a representative HIV-1-infected individual after stimulation with the corresponding peptide. PBMCs from HIV-1-infected individuals were incubated with the ovalbumin (OVA)_257−264_ peptide (negative peptide, abbreviated as NP), SL9, IL9, or TL9 (2 *μ*M) and purified anti-CD28 antibody (1 *μ*g/ml) and recombinant IL-2 (20 ng/ml) for 6 hours at 37°C, in the presence of brefeldin A (2 *μ*g/ml) for the final 2 hours of incubation. The numbers represent the percentages of CD95^+^CD122^+^ T_SCM_s producing IFN-*γ*. The data are representative of six independent experiments (*n* = 6). (d) The percentages of CD8^+^ T_SCM_ subsets producing IFN-*γ*; data from 20 HIV-1-infected individuals are shown as the mean ± SEM. The Kruskal–Wallis test with Dunn's multiple comparisons was performed to assess statistical significance. ^∗∗^ *P* < 0.01. (e) The mean fluorescence intensities of CD8^+^ T_SCM_ subsets producing IFN-*γ* are shown as the mean ± SEM. The Kruskal–Wallis test with Dunn's multiple comparisons was performed to assess statistical significance. The data are representative of four independent experiments (*n* = 6). ^∗∗^ *P* < 0.01.

**Figure 2:**
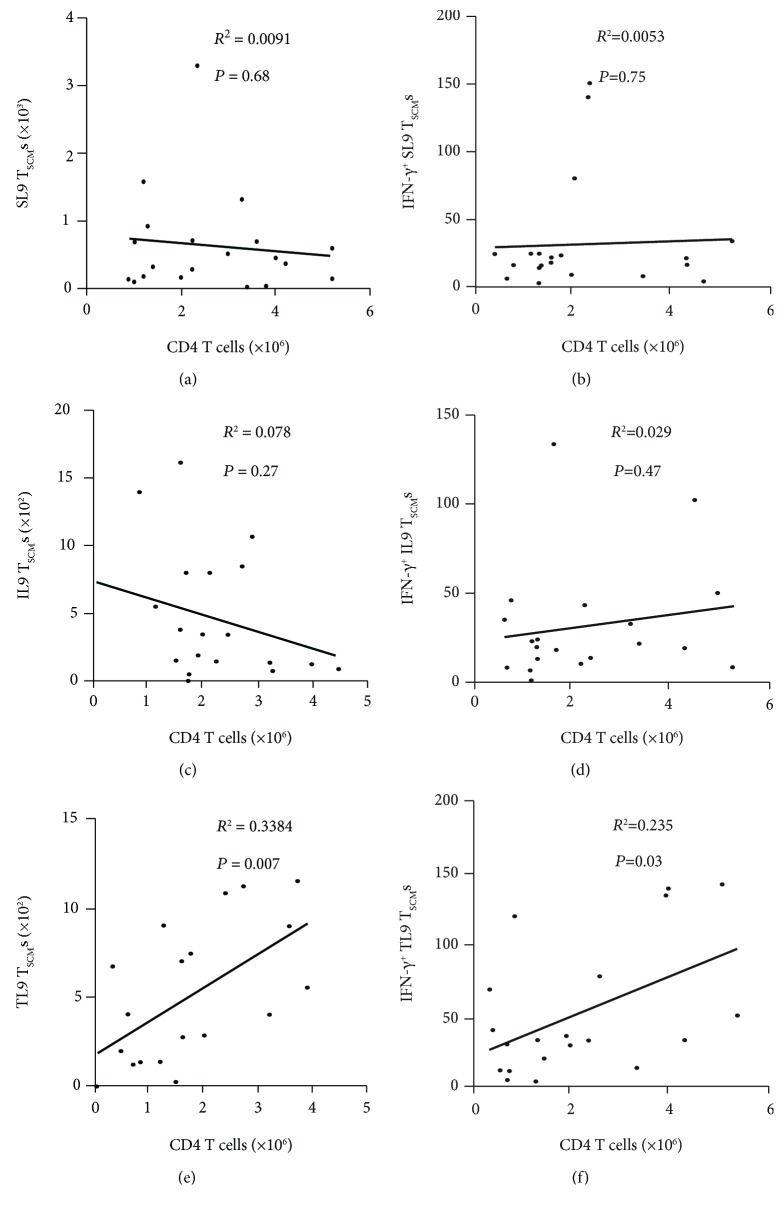
Associations between epitope-specific CD8^+^ T memory stem cells and CD4^+^ T cell counts in the peripheral blood of HIV-1-infected individuals. (a–c) The association between CD4^+^ T cell counts and absolute counts of HIV-1-specific CD8^+^ T memory stem cells (T_SCM_s) in 20 combined antiretroviral therapy- (cART-) treated HIV-1-infected individuals. Spearman's rank correlation coefficients are shown. (d–f) Associations between CD4^+^ T cell counts and the proportions of interferon gamma- (IFN-*γ*-) secreting HIV-1-specific CD8^+^ T_SCM_s from cART-treated HIV-1-infected individuals. Spearman's rank correlation coefficients are shown.

**Figure 3:**
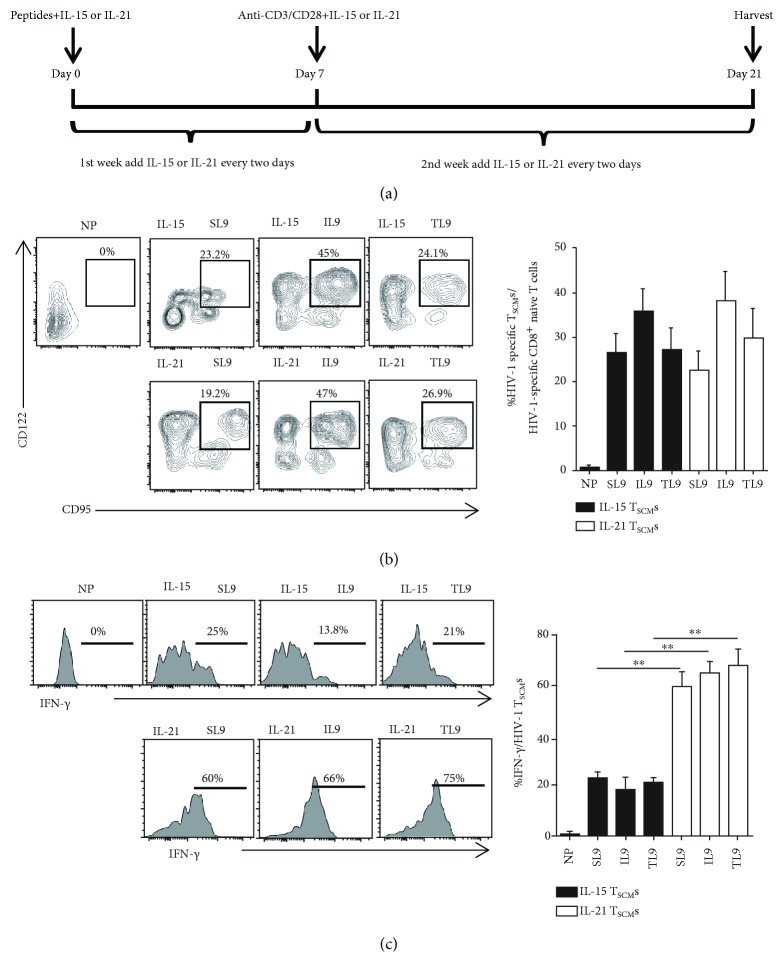
Interleukin-21 efficiently induces the production of epitope-specific CD8^+^ T memory stem cells. (a) Schematic diagram of the generation of HIV-1 antigen-specific CD8^+^ T memory stem cells (T_SCM_s). Naïve CD8^+^ T cells (CD3^+^CD8^+^CD45RA^+^CD45RO^−^CD62L^+^CCR7^+^CD95^−^CD122^−^) were sorted from the PBMCs of HIV-1-infected individuals, and then, the cells (1 × 10^6^/ml) were mixed with irradiated autologous antigen-presenting cells (APCs; 5 × 10^6^/ml). The cell mixtures were stimulated with a peptide cocktail (each peptide at a concentration of 2 *μ*M; ovalbumin [OVA]_257–264_ peptide alone served as the negative control peptide, abbreviated as NP) with interleukin- (IL-) 21 (20 ng/ml) or IL-15 (20 ng/ml) for 7 days. The medium and cytokines were refreshed every 2 days. After 1 week, the cell mixtures were stimulated with anti-CD3 (2 *μ*g/ml) and anti-CD28 (1 *μ*g/ml) antibodies with IL-21 (20 ng/ml) or IL-15 (20 ng/ml) for 14 days. The medium and cytokines were refreshed every 2 days. (b) Flow cytometric analysis of the frequencies of antigen-specific CD8^+^ T_SCM_s that were expanded from HIV-1-infected individuals in the presence of IL-15 (top panel) or IL-21 (bottom panel). Dot plots show the identification of HIV-1 antigen-specific CD95^+^CD122^+^ T_SCM_s (based on a tetramer^+^CD8^+^CD45RA^+^CD45RO^−^CD62L^+^CCR7^+^ phenotype for T_SCM_s stimulated with specific peptides or a CD8^+^CD45RA^+^CD45RO^−^CD62L^+^CCR7^+^ phenotype for T_SCM_s stimulated with NP). The numbers in the graphs show the percentages of CD8^+^ T_SCM_s after each treatment. The Kruskal–Wallis test with Dunn's multiple comparisons was performed to assess statistical significance. The data are shown as the mean ± standard error of the mean (SEM) from five independent experiments. (c) Cytokine secretion profiles of HIV-1-specific CD8^+^ T_SCM_s generated with IL-15 (top panel) or IL-21 (bottom panel). The CD8^+^ T_SCM_s sorted by flow cytometry were cocultured with irradiated autologous APCs. Cell mixtures were incubated with NP, SL9, IL9, or TL9 (2 *μ*M) and purified anti-CD28 antibody (1 *μ*g/ml) and recombinant IL-2 (20 ng/ml) for 6 hours at 37°C, in the presence of brefeldin A (2 *μ*g/ml) for the final 2 hours of incubation. The numbers in the graphs show the percentages of IFN-*γ* ^+^ cells among tetramer^+^CD8^+^ T_SCM_s after each treatment. The Kruskal–Wallis test with Dunn's multiple comparisons was performed to assess statistical significance. The data are shown as the mean ± SEM of five independent experiments. ^∗∗^ *P* < 0.01.

PBMCs were separated from whole blood by density gradient centrifugation with Ficoll Paque, and CD8^+^ T_SCM_s (CD8^+^CD45RA^+^CD45RO^−^CD62L^+^CCR7^+^CD95^+^CD122^+^) were sorted by flow cytometry. For Figures 1 and 2, PBMCs were incubated with appropriate peptides (2 *μ*M) at 37°C in RPMI 1640 GlutaMAX™-I medium (Gibco™, Life Technologies, Carlsbad, CA, USA) containing 9% fetal bovine serum (FBS; Gibco™, Life Technologies, Carlsbad, CA, USA), penicillin (100 U/ml; HyClone, GE Healthcare Life Sciences, Chicago, IL, USA), streptomycin (100 *μ*g/ml; HyClone, GE Healthcare Life Sciences, Chicago, IL, USA), and IL-2 (20 ng/ml; R&D Systems Minneapolis, MN). For Figure 3, autologous antigen-presenting cells (APCs) were prepared from PBMCs. After the depletion of total T cells with anti-CD3 antibody-coated beads (Miltenyi Biotec, Bergisch Gladbach, Germany), the PBMCs were washed, irradiated (10 Gy), and plated (5 × 10^5^ cells/well) in 96-well cell culture plates (Costar™, Corning™, Corning, NY, USA). The resulting APCs were incubated at 37°C in RPMI 1640 GlutaMAX™-Ι medium for 4 hours in the presence of the appropriate peptides (2 *μ*M). CD8^+^ T_SCM_s were seeded over the pulse-irradiated autologous APCs (5 × 10^5^ cells/well) in culture medium containing 10% FBS and human IL-2 (20 ng/ml; R&D Systems, Minneapolis, MN, USA). The T cell populations were harvested and stained for flow cytometric analysis using BD LSRFortessa™ (BD Biosciences, San Jose, CA, USA).

### 2.4. Cytokine Detection

Intracellular cytokine production was assessed as previously described [26]. Briefly, PBMCs were incubated with specific peptides (SL9 (SLYNTVATL), IL9 (ILKEPVHGV), and TL9 (TLNAWVLVV); 2 *μ*M), respectively, or ovalbumin 257–264 (SIINFEKL; as a negative control), purified anti-CD28 antibody (1 *μ*g/ml; BD Pharmingen, San Jose, CA, USA), and recombinant IL-2 (20 ng/ml; R&D Systems Minneapolis, MN) for 6 hours at 37°C and in the presence of brefeldin A (2 *μ*g/ml; Sigma–Aldrich, St. Louis, MO, USA) for the final 2 hours of incubation. Cells were stained with PE-conjugated tetramer for 20 min at 37°C before staining for surface antibodies. Cell surface staining was completed as described after *in vitro* activation for 6 hours [27]. Cells were then permeabilized with Cytofix/Cytoperm™ (BD Pharmingen, San Jose, CA, USA) and labeled with APC-conjugated anti-human IFN-*γ* (IgG1, B27; eBioscience, San Diego, CA, USA).

### 2.5. Transwell Viral Outgrowth Assays

Transwell viral outgrowth assays were performed as previously described [24, 28, 29]. Briefly, we depleted CD8^+^ T cells from the PBMCs isolated from HIV-1-infected individuals with human CD8 microbeads (Miltenyi Biotec, Bergisch Gladbach, Germany) and then activated the cells with phorbol 12-myristate 13-acetate (PMA; 500 ng/ml; Sigma-Aldrich, St. Louis, MO, USA) and ionomycin (1 *μ*g/ml; Sigma-Aldrich, St. Louis, MO, USA) in the presence of IL-2 (20 ng/ml; R&D Systems, Minneapolis, MN, USA) for 1 week. To promote virus release from infected cells, we prepared CD4^+^ T lymphoblasts 2–3 days before adding them to culture assays, rendering them fully tolerant to viral infection at the time of addition. The CD4^+^ T lymphoblasts from healthy donors were activated with phytohemagglutinin (PHA; 5 *μ*g/ml; Sigma-Aldrich, St. Louis, MO, USA) and IL-2 (20 ng/ml) and then cocultured with patient cells for 3 days (for PCR assays) or 14 days (for enzyme-linked immunosorbent assays (ELISAs)). The two populations of cells were separated by a cell-impermeable polyester membrane with 0.4 *μ*m pores (Costar™, Corning™, Corning, NY, USA).

### 2.6. p24 ELISA

The concentrations of p24 in the cell supernatants were determined by ELISA with the HIV-1 p24 ELISA Kit (Clontech, Takara Bio USA, Mountain View, CA, USA), according to the manufacturer's instructions [30, 31].

### 2.7. Generation of HIV-1 Antigen-Specific CD8^+^ T_SCM_s *In Vitro*

Autologous APCs were prepared from the PBMCs of HIV-1-infected individuals after the depletion of CD3^+^ T cells with human CD3 microbeads (Miltenyi Biotec, Bergisch Gladbach, Germany). Then, APCs were irradiated (10 Gy) and plated (5 × 10^6^ cells/well) in 12-well cell culture plates (Costar™, Corning™, Corning, NY, USA). The irradiated APCs were incubated at 37°C in RPMI 1640 GlutaMAX™-I medium in the presence of SL9, IL9, and TL9 peptides (2 *μ*M). CD8^+^CD45RA^+^CD45RO^−^CCR7^+^CD62L^+^CD122^−^CD95^−^ naïve T cells (1 × 10^6^) were cocultured with APCs in the presence of IL-21 or IL-15 (20 ng/ml; R&D Systems, Minneapolis, MN, USA). After 7 days, the cells were stimulated with anti-CD3 (2 *μ*g/ml; BD Pharmingen, San Jose, CA, USA), anti-CD28 (1 *μ*g/ml; BD Pharmingen, San Jose, CA, USA), and IL-21 or IL-15 (20 ng/ml, R&D Systems, Minneapolis, MN, USA). CD8^+^ T_SCM_s (CD8^+^CD45RA^+^CD45RO^−^CD62L^+^CCR7^+^CD95^+^CD122^+^) were sorted by flow cytometry, from mixed cells (BD FACSAria™ II, BD Biosciences, San Jose, CA, USA). The data were analyzed with FlowJo® (FlowJo, Treestar Inc., San Carlos, CA). Antibodies used for flow cytometry are listed in Supplementary Table II.

### 2.8. Statistical Analysis

We used the Kruskal–Wallis test with Dunn's multiple comparisons to compare three unpaired sample groups and Friedman's test with Dunn's multiple comparisons for the comparison of three paired sample groups.

## 3. Results

### 3.1. HIV-1-Specific T_SCM_s Are Present in Patient PBMCs

The specificity of CD8^+^ T cells to pathogens is dependent on 8–12 amino acid epitopes that are presented by corresponding major histocompatibility complex class I molecules [32]. Although the existence of CD8^+^ T_SMC_s in HIV-1-infected individuals has been demonstrated, their specificity for unmutated antigen epitopes, especially those that are broadly expressed, has not yet been well characterized [11, 33]. To identify CD8^+^ T_SCM_s specific for unmutated antigen epitopes, which could be suitable for HIV-1 immunotherapy to eradicate viral reservoirs, we exploited tetramers and analyzed the frequencies of CD8^+^ T_SCM_s specific for each epitope in the peripheral blood of HIV-1-infected individuals. We chose three well-characterized CTL epitopes as follows: the HLA-A2-restricted epitopes SLYNTVATL (SL9), ILKEPVHGV (IL9), and TLNAWVLVV (TL9). Based on previous reports, a highly stringent phenotype for T_SCM_s, based on seven surface markers (CD8^+^, CD45RA^+^, CD45RO^−^, CD62L^+^, CCR7^+^, CD95^+^, and IL-2R*β* ^+^), was used to accurately analyze the proportion of cells specific for each epitope, and twenty HIV-1-infected individuals were recruited for T_SCM_ analysis (Supplemental Table I) [7]. In the peripheral blood of HIV-1-infected individuals, CD8^+^ T_SCM_s that recognized three HIV-1 antigenic epitopes were identified by flow cytometric analysis (Figure 1(a), Figures S1 and S2). To exclude contamination by other cell subsets (i.e., NK cells, B cells, and monocytes) and nonspecific CD8^+^ T_SCM_s, we also stained mismatched tetramer (nonspecific tetramer binding), CD56 (NK cells), CD14 (monocytes), and CD19 (B cells) on the surfaces of CD8^+^ T_SCM_s from individuals infected with HIV-1. NK cells, B cells, or monocytes among the CD8^+^ T_SCM_s were not detected, unlike results from PBMCs (positive controls for the antibodies), and the antigen-specific CD8^+^ T_SCM_s that we identified in HIV-1-infected individuals were confirmed to be HIV-1-specific (Figures S3 and S4). The absolute numbers of the CD8^+^ T_SCM_s specific for the three epitopes were almost equal (Figure 1(b)). To analyze the functional properties of the HIV-1-specific CD8^+^ T_SCM_s, we assayed the production of IFN-*γ* in these cells (Figure 1(c)). Consistent with previous findings, all HIV-1 antigen-specific T_SCM_s secreted IFN-*γ* upon exposure to corresponding peptides (Figure 1(c)). Interestingly, despite the equal proportions of IFN-*γ* ^+^ cells in each group, the mean fluorescence intensity of the IFN-*γ* signal in TL9-specific CD8^+^ T_SCM_s was higher than that in SL9- or IL9-specific CD8^+^ T_SCM_s, indicating the increased production of IFN-*γ* per cell (Figures 1(d) and 1(e)). The TL9 epitope is an unmutated CTL epitope, and thus, CTLs specific for TL9 would possess broad antiviral capability to effectively control latent infection with a variety of HIV-1 strains [2]. Taken together, our data demonstrate the existence of functional HIV-1 antigen-specific CD8^+^ T_SCM_s in the blood of HIV-1-infected individuals.

### 3.2. Associations between Epitope-Specific CD8^+^ T_SCM_s in the Blood and Clinical HIV-1 Disease Progression

To evaluate the possible influence of each epitope-specific CD8^+^ T_SCM_ population on the clinical parameters of HIV-1 disease progression during infection, a possible correlation between the absolute number of HIV-1 epitope-specific CD8^+^ T_SCM_s and the number of CD4^+^ T cells was analyzed. We found that the number of CD4^+^ T lymphocytes correlated with the number of TL9-specific CD8^+^ T_SCM_s, but not with the number of SL9- or IL9-specific CD8^+^ T_SCM_s (Figure 2(a)). Moreover, there was a similar association between the number of CD4^+^ T cells and IFN-*γ* secretion in each epitope-specific CD8^+^ T_SCM_ population (Figure 2(b)). These data indicate that CD8^+^ T_SCM_s specific for TL9 might play a key role in immune surveillance in response to HIV-1 replication.

### 3.3. IL-21 Efficiently Expands Epitope-Specific CD8^+^ T_SCM_s

We next sought to induce the generation of HIV-1-specific CD8^+^ T_SCM_s via IL-21 or IL-15 treatment (Figure 3(a)). Given that the HIV-1 antigen-specific CD8^+^ T_SCM_s appeared to be unable to survive or proliferate during ongoing viral replication in untreated HIV-1-infected individuals and to avoid interference by endogenous HIV-1-specific CD8^+^ T_SCM_s, we first isolated highly purified naïve CD8^+^ T cells. Subsequently, CD8^+^ T_SCM_s were generated *in vitro* [2, 8, 33]. Similar to that observed after treatment with IL-15, IL-21 treatment expanded each population of epitope-specific CD8^+^ T_SCM_s (Figure 3(b), Figure S5). Importantly, after 3 days of exposure to corresponding antigens, CD8^+^ T_SCM_s secreted higher levels of IFN-*γ* after culture with IL-21, compared to that with IL-15 (Figure 3(c)). These results indicated that IL-21-induced HIV-1-specific CD8^+^ T_SCM_s might possess robust capacity to control HIV-1 replication.

### 3.4. IL-21-Generated CD8^+^ T_SCM_s Suppress HIV-1 Replication

**Figure 4:**
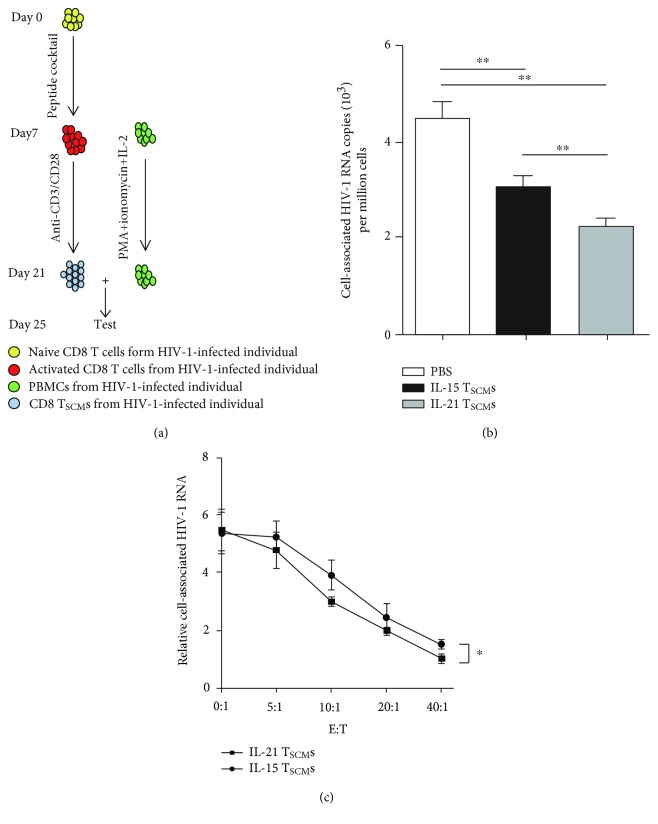
IL-21-generated CD8^+^ T memory stem cells suppress HIV-1 production. (a) Schematic diagram of the suppression assay for *in vitro*-generated CD8^+^ T_SCM_s. Naïve CD8^+^ T cells (CD3^+^CD8^+^CD45RA^+^CD45RO^−^CD62L^+^CCR7^+^CD95^−^CD122^−^) were sorted from the PBMCs of HIV-1-infected individuals, and then, the cells (1 × 10^6^/ml) were mixed with irradiated autologous antigen-presenting cells (APCs; 5 × 10^6^/ml). The cell mixtures were stimulated with a peptide cocktail (each peptide at a concentration of 2 *μ*M) with interleukin- (IL-) 21 (20 ng/ml) or IL-15 (20 ng/ml) for 7 days. The medium and cytokines were refreshed every 2 days. After 1 week, the cell mixtures were stimulated with anti-CD3 (2 *μ*g/ml) and anti-CD28 (1 *μ*g/ml) antibodies with IL-21 (20 ng/ml) or IL-15 (20 ng/ml) for 14 days. The medium and cytokines were refreshed every 2 days. After 14 days, polyclonal CD8^+^ T_SCM_s (1 × 10^6^) were then sorted by flow cytometry. Autologous CD8^+^ T-lymphocyte-depleted PBMCs (2 × 10^5^) that were activated with PMA (500 ng/ml) and ionomycin (1 *μ*g/ml) plus with IL-2 (20 ng/ml) for 14 days and then cocultured with CD8^+^ T_SCM_s. After 4 days of coculture, the HIV-1 production was tested by qRT-PCR. (b) HIV-1 replication was measured by quantitative reverse transcription polymerase chain reaction (qRT-PCR). After various treatments for 4 days, HIV-1 viral production was determined by measuring cell-associated HIV-1 RNA by qRT-PCR (10^3^ copies/1 × 10^6^ cells). The data are shown as the mean ± standard error of the mean (SEM) of four independent experiments (*n* = 6). The Kruskal–Wallis test with Dunn's multiple comparisons was performed to assess statistical significance. ^∗∗^ *P* < 0.01. (c) The antiviral capacity of CD8^+^ T_SCM_s was dose-dependent. Purified CD8^+^ T_SCM_s (effector cells, abbreviated as E) were cocultured with autologous CD8^+^ T-lymphocyte-depleted PBMCs (target cells, abbreviated as T). After treatment for 3 days, HIV-1 production was determined by relative quantification of cell-associated HIV-1 RNA normalized to a CD4 gene level. The data are shown as the mean ± SEM of four independent experiments (*n* = 4). The slopes were compared using a Wilcoxon rank test. ^∗^ *P* < 0.05, ^∗∗^ *P* < 0.01.

To assess the antiviral effects of IL-21-generated CD8^+^ T_SCM_s, we tested the suppressive function of T_SCM_s generated *in vitro* on HIV-1 production in the presence of various cytokines (Figure 4(a)) [8]. As it is not practical to purify HIV-1 antigen-specific CD8^+^ T_SCM_s for this assay, we sorted total polyclonal CD8^+^ T_SCM_s (CD8^+^CD45RA^+^CD45RO^−^CD62L^+^CCR7^+^CD95^+^CD122^+^ T cells) by flow cytometry and cocultured these cells with autologous CD8^+^ T lymphocyte-depleted PBMCs (Figure S6). We then performed real-time qRT-PCR to analyze cell-associated HIV-1 RNA in the autologous PBMCs 4 days later. Compared to that observed upon treatment with the vehicle-cultured CD8^+^ T_SCM_s, the administration of IL-15- or IL-21-generated CD8^+^ T_SCM_s resulted in considerably lower levels of cell-associated HIV-1 RNA in the PBMCs (Figure 4(b)). Furthermore, the inhibitory effect of IL-21-generated CD8^+^ T_SCM_s on HIV-1 RNA was even greater than that of IL-15-generated CD8^+^ T_SCM_s (Figure 4(b)). As expected, the suppressive capacity of CD8^+^ T_SCM_s was dose-dependent (Figure 4(c)). Taken together, these data indicate that IL-21-generated CD8^+^ T_SCM_s effectively suppress HIV-1 production.

### 3.5. IL-21-Generated CD8^+^ T_SCM_s Suppress HIV-1 Production More Efficiently than Conventional CD8^+^ Memory Cells

**Figure 5:**
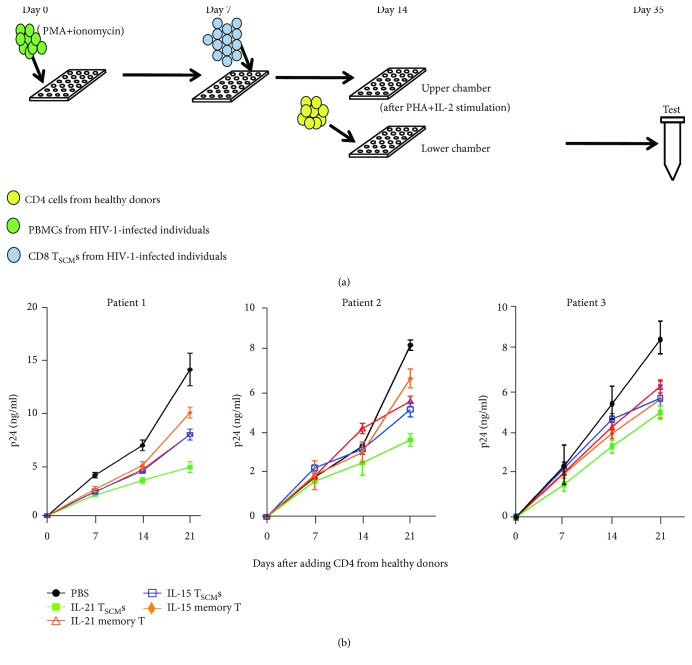
More robust suppression of HIV-1 production with IL-21-generated CD8^+^ T memory stem cells. (a) Schematic diagram of the transwell suppression assay for *in vitro*-generated CD8^+^ T memory stem cells (T_SCM_s). Naïve CD8^+^ T cells (CD3^+^CD8^+^CD45RA^+^CD45RO^−^CD62L^+^CCR7^+^CD95^−^CD122^−^) were sorted from the PBMCs of HIV-1-infected individuals, and then, the cells (1 × 10^6^/ml) were mixed with irradiated autologous antigen-presenting cells (APCs; 5 × 10^6^/ml). The cell mixtures were stimulated with a peptide cocktail (each peptide at a concentration of 2 *μ*M) with interleukin- (IL-) 21 (20 ng/ml) or IL-15 (20 ng/ml) for 7 days. The medium and cytokines were refreshed every 2 days. After 1 week, the cell mixtures were stimulated with anti-CD3 (2 *μ*g/ml) and anti-CD28 (1 *μ*g/ml) antibodies with IL-21 (20 ng/ml) or IL-15 (20 ng/ml) for 14 days. The medium and cytokines were refreshed every 2 days. The polyclonal CD8^+^ T_SCM_s (1 × 10^6^) were then sorted and cocultured with autologous CD8^+^ T-lymphocyte-depleted PBMCs (2 × 10^5^) that were activated with PMA (500 ng/ml) and ionomycin (1 *μ*g/ml) in the presence of IL-2 (20 ng/ml). After 1 week, the mixtures were moved to the upper chambers of a transwell system and fresh CD4^+^ T cells, isolated from healthy donors, were activated with phytohemagglutinin (0.5 *μ*g/ml) and IL-2 (20 ng/ml) and added to the lower chamber. The concentrations of HIV-1 p24 in the supernatants were tested by ELISA. (b) The inhibitory effect of CD8^+^ T_SCM_s on the replication of HIV-1. Viral replication was monitored by the continuous detection of HIV-1 p24 antigen in the supernatant of each treatment group by ELISA. The data are shown as the mean ± standard error of the mean of three independent experiments (*n* = 3).

Conventional memory T cells can eliminate various pathogens [34]. However, it remains unknown if the inhibitory effect of CD8^+^ T_SCM_s on HIV-1 replication is superior to that of conventional memory CD8^+^ T cells. Given the excellent performance of CD8^+^ T_SCM_s in melanoma, we speculated that they could also achieve better HIV-1 suppression than conventional memory T cells [35]. To test our hypothesis, we isolated conventional CD8^+^ memory T cells (CD8^+^CD45RA^−^CD45RO^+^) and CD8^+^ T_SCM_s and then performed cocultures with autologous PBMCs. Meanwhile, we used a transwell culture system to prevent HIV-1 antigen-specific CD8^+^ T_SCM_s from mediating cytotoxicity against exogenous healthy donor PBMCs (Figure 5(a)) [24]. The viral suppressive capacity of each T cell subset was assessed by detecting HIV-1 p24 antigen in the culture supernatants (Figure 5(a)). As expected, the production of p24 in cocultures with IL-21-generated CD8^+^ T_SCM_s was lower than that in cocultures with IL-21-generated conventional CD8^+^ memory T cells or IL-15-generated CD8^+^ T_SCM_s (Figure 5(b)). Our data indicate that IL-21-generated CD8^+^ T_SCM_s possess greater viral suppressive capacity than conventional memory CD8^+^ T cells.

## 4. Discussion

According to previous reports, documented CTL escape variants dominate the viral reservoirs of almost all HIV-1-infected individuals in the chronic phase [2, 36, 37]. This trend is particularly obvious for some well-characterized CTL epitopes including HLA-A2-restricted SLYNTVATL (SL9), HLA-A3-restricted RLRPGGKKK (RK9), and HLA-B57/58-restricted TSTLQEQIGW (TW10). Previously, the characterization of CD8^+^ T_SCM_s in HIV-1-infected individuals was restricted to these mutant epitopes [33, 38]. Therefore, the utilization of these epitopes results in failure for long-term immunotherapies [2, 39]. In our study, we explored the abundance of HIV-1 antigen-specific CD8^+^ T_SCM_s that recognize unmutated CTL epitopes and compared their immune competence with those recognizing other epitopes. Despite similar numbers of antigen-specific cells and IFN-*γ* ^+^ cells among each population of epitope-specific CD8^+^ T_SCM_s, the mean IFN-*γ* secretion by TL9-specific CD8^+^ T_SCM_s was higher. Our data also revealed that the proportions of TL9-specific CD8^+^ T_SCM_s and IFN-*γ* ^+^ TL9-specific CD8^+^ T_SCM_s positively correlated with CD4^+^ T cell counts. These data are consistent with the previous observation that targeting broad CTL epitopes is essential for the clearance of HIV-1 latently infected cells and demonstrate the feasibility of utilizing broad CTL epitopes for the expansion of HIV-1 antigen-specific CD8^+^ T_SCM_s [2]. Unfortunately, due to the limited availability of tetramers, the roles of other broadly unmutated epitopes in HIV-1 antigen-specific CD8^+^ T_SCM_ responses remain unknown. Simultaneously, due to the lack of adequate cell numbers from each clinical sample, we only measured the functional properties of HIV-1 antigen-specific CD8^+^ T_SCM_s based on their expression of IFN-*γ*. It remains unknown whether the production of other cytotoxic factors such as granzyme B, tumor necrosis factor alpha, and perforin is also higher in HIV-1 antigen-specific CD8^+^ T_SCM_s than in conventional memory CD8^+^ T cells.

Given the convincing performance of CD8^+^ T_SCM_ adoptive transfer therapy for other chronic diseases, we utilized relevant peptides to expand HIV-1 antigen-specific CD8^+^ T_SCM_s. According to a previous report, although SL9 and IL9 are frequently mutated epitopes, they are abundantly found in the peripheral blood of HIV-1-infected individuals and they stimulate high levels of IFN-*γ* production prior to mutation [2]. Based on this previous study, we also applied SL9 and IL9 for the expansion of HIV-1 antigen-specific CD8^+^ T_SCM_s. Given that the vast majority of genes are expressed similarly in conventional memory T cells and T_SCM_s and that IL-21 can efficiently induce the generation of conventional memory T cells, it is necessary to explore IL-21 as a potential cytokine for the induction of T_SCM_s. We found that IL-21 efficiently stimulated the expansion of T_SCM_s [7]. Surprisingly, although IL-21 and IL-15 similarly stimulated the expansion of HIV-1 antigen-specific CD8^+^ T_SCM_s, based on cell numbers, IL-21 resulted in enhanced expression of IFN-*γ* compared to that with IL-15. Consistent with this effect, CD8^+^ T_SCM_s stimulated with IL-21 mediated more robust viral suppression. Notably, unlike the effects of broad CTLs, the antiviral effect of CD8^+^ T_SCM_s (as assessed by the abundance of cell-associated viral RNA) was not significantly enhanced when the PBMCs of HIV-1-infected individuals were mixed with autologous CD8^+^ T cells at a 1 : 1 ratio (data not shown). We speculate that although CD8^+^ T_SCM_s efficiently proliferated and survived, their response might be slower than that of previously tested effector T cells. Due to the limited number of HIV-1-specific CD8^+^ T_SCM_s, we did not isolate high-purity tetramer^+^ cells, which could account for the relatively low cytotoxicity. Therefore, we could not detect significantly higher antiviral effects of CD8^+^ T_SCM_s over 3 days, based on p24 levels. Taken together, our data have expanded our knowledge regarding the use of IL-21 for antiviral therapy. However, whether the administration of recombinant IL-21 can directly facilitate the generation of HIV-1 antigen-specific CD8^+^ T_SCM_s *in vivo* needs to be clarified. Certainly, it is obvious that IL-21-generated CD8^+^ T_SCM_ titers might not be very high *in vivo*; therefore, we speculated that IL-21-induced CD8^+^ T_SCM_s exert their antiviral effect by differentiating into other T cell subsets to suppress the virus replication.

Overall, our study demonstrated the presence of immune-competent HIV-1 antigen-specific CD8^+^ T_SCM_s that recognize unmutated epitopes in the peripheral blood of HIV-1-infected individuals, as well as the efficient generation of these cells *in vitro* in response to IL-21. We also demonstrated that the immunologic activity of IL-21-generated CD8^+^ T_SCM_s against HIV-1 is greater than that of IL-15-generated CD8^+^ T_SCM_s. Our study provides a novel insight into the antiviral effects of T_SCM_s in HIV-1-infected individuals and the potential role for IL-21 in antiviral immunotherapy against HIV-1.

## Data Availability

The clinical sample data used to support the findings of this study are included within the article.

## Supplementary Materials

Supplementary Materials Table I: summary of HIV-1-infected individuals. Table II: antibodies used for flow cytometry. Figure S1: gating strategy for live cells (the middle panel) and single cells (right panel). Figure S2: the Fluorescence Minus One (FMO) controls for flow cytometric analysis of HIV-1 antigen-specific CD8^+^ TSCMs. Figure S3: the detection of nonspecific tetramer binding. Figure S4: expression of CD19, CD14, and CD56 in CD8^+^ T memory stem cells (TSCMs) and peripheral blood mononuclear cells (PBMCs). Figure S5: gating strategy for the analysis of in vitro-generated CD8^+^ T memory stem cells (TSCMs). Figure S6: purity of CD3^+^CD8^+^CD45RO^−^CD45RA^+^CD62L^+^CCR7^+^CD95^+^CD122^+^ T cells after sorting by flow cytometry.

## References

[1] Deeks S. G., Lewin S. R., Havlir D. V. (2013). The end of AIDS: HIV infection as a chronic disease. *The Lancet*.

[2] Deng K., Pertea M., Rongvaux A. (2015). Broad CTL response is required to clear latent HIV-1 due to dominance of escape mutations. *Nature*.

[3] Siliciano J. D., Kajdas J., Finzi D. (2003). Long-term follow-up studies confirm the stability of the latent reservoir for HIV-1 in resting CD4+ T cells. *Nature Medicine*.

[4] Strain M. C., Gunthard H. F., Havlir D. V. (2003). Heterogeneous clearance rates of long-lived lymphocytes infected with HIV: intrinsic stability predicts lifelong persistence. *Proceedings of the National Academy of Sciences of the United States of America*.

[5] Lu W., Arraes L. C., Ferreira W. T., Andrieu J. M. (2004). Therapeutic dendritic-cell vaccine for chronic HIV-1 infection. *Nature Medicine*.

[6] Collins K. L., Chen B. K., Kalams S. A., Walker B. D., Baltimore D. (1998). HIV-1 Nef protein protects infected primary cells against killing by cytotoxic T lymphocytes. *Nature*.

[7] Gattinoni L., Lugli E., Ji Y. (2011). A human memory T cell subset with stem cell-like properties. *Nature Medicine*.

[8] Cieri N., Camisa B., Cocchiarella F. (2013). IL-7 and IL-15 instruct the generation of human memory stem T cells from naive precursors. *Blood*.

[9] Lugli E., Gattinoni L., Roberto A. (2013). Identification, isolation and in vitro expansion of human and nonhuman primate T stem cell memory cells. *Nature Protocols*.

[10] Papatriantafyllou M. (2011). T cell memory: the stem of T cell memory. *Nature Reviews Immunology*.

[11] Ribeiro S. P., Milush J. M., Cunha-Neto E. (2014). The CD8+ memory stem T cell (TSCM) subset is associated with improved prognosis in chronic HIV-1 infection. *Journal of Virology*.

[12] Mateus J., Lasso P., Pavia P. (2015). Low frequency of circulating CD8+ T stem cell memory cells in chronic chagasic patients with severe forms of the disease. *PLoS Neglected Tropical Diseases*.

[13] Gattinoni L., Restifo N. P. (2013). Moving T memory stem cells to the clinic. *Blood*.

[14] Ozaki K., Spolski R., Feng C. G. (2002). A critical role for IL-21 in regulating immunoglobulin production. *Science*.

[15] Rochman Y., Spolski R., Leonard W. J. (2009). New insights into the regulation of T cells by gamma(c) family cytokines. *Nature reviews Immunology*.

[16] Giri J. G., Ahdieh M., Eisenman J. (1994). Utilization of the beta and gamma chains of the IL-2 receptor by the novel cytokine IL-15. *The EMBO Journal*.

[17] Spolski R., Leonard W. J. (2014). Interleukin-21: a double-edged sword with therapeutic potential. *Nature reviews Drug Discovery*.

[18] Sabatino M., Hu J., Sommariva M. (2016). Generation of clinical-grade CD19-specific CAR-modified CD8+ memory stem cells for the treatment of human B-cell malignancies. *Blood*.

[19] White L., Krishnan S., Strbo N. (2007). Differential effects of IL-21 and IL-15 on perforin expression, lysosomal degranulation, and proliferation in CD8 T cells of patients with human immunodeficiency virus-1 (HIV). *Blood*.

[20] Chevalier M. F., Julg B., Pyo A. (2011). HIV-1-specific interleukin-21^+^ CD4^+^ T cell responses contribute to durable viral control through the modulation of HIV-specific CD8^+^ T cell function. *Journal of Virology*.

[21] Adoro S., Cubillos-Ruiz J. R., Chen X. (2015). IL-21 induces antiviral microRNA-29 in CD4 T cells to limit HIV-1 infection. *Nature Communications*.

[22] Williams L. D., Amatya N., Bansal A. (2014). Immune activation is associated with CD8 T cell interleukin-21 production in HIV-1-infected individuals. *Journal of Virology*.

[23] Agosto L. M., Yu J. J., Dai J., Kaletsky R., Monie D., O'Doherty U. (2007). HIV-1 integrates into resting CD4+ T cells even at low inoculums as demonstrated with an improved assay for HIV-1 integration. *Virology*.

[24] Ho Y. C., Shan L., Hosmane N. N. (2013). Replication-competent noninduced proviruses in the latent reservoir increase barrier to HIV-1 cure. *Cell*.

[25] Zhang Y., Yin Y., Zhang S., Luo H., Zhang H. (2016). HIV-1 infection-induced suppression of the let-7i/IL-2 axis contributes to CD4(+) T cell death. *Scientific Reports*.

[26] Horton H., Thomas E. P., Stucky J. A. (2007). Optimization and validation of an 8-color intracellular cytokine staining (ICS) assay to quantify antigen-specific T cells induced by vaccination. *Journal of Immunological Methods*.

[27] Northfield J. W., Kasprowicz V., Lucas M. (2008). CD161 expression on hepatitis C virus-specific CD8+ T cells suggests a distinct pathway of T cell differentiation. *Hepatology*.

[28] Siliciano J. D., Siliciano R. F., Zhu T. (2005). Enhanced culture assay for detection and quantitation of latently infected, resting CD4^+^ T-cells carrying replication-competent virus in HIV-1-infected individuals. *Human Retrovirus Protocols*.

[29] Liu B., Zou F., Lu L. (2016). Chimeric antigen receptor T cells guided by the single-chain Fv of a broadly neutralizing antibody specifically and effectively eradicate virus reactivated from latency in CD4+ T lymphocytes isolated from HIV-1-infected individuals receiving suppressive combined antiretroviral therapy. *Journal of Virology*.

[30] Huang F., Zhang J., Zhang Y. (2015). RNA helicase MOV10 functions as a co-factor of HIV-1 Rev to facilitate Rev/RRE-dependent nuclear export of viral mRNAs. *Virology*.

[31] Geng G., Liu B., Chen C. (2016). Development of an attenuated tat protein as a highly-effective agent to specifically activate HIV-1 latency. *Molecular Therapy*.

[32] Neefjes J., Ovaa H. (2013). A peptide’s perspective on antigen presentation to the immune system. *Nature Chemical Biology*.

[33] Vigano S., Negron J., Ouyang Z. (2015). Prolonged antiretroviral therapy preserves HIV-1-specific CD8 T cells with stem cell-like properties. *Journal of Virology*.

[34] Bajénoff M., Narni-Mancinelli E., Brau F., Lauvau G. (2010). Visualizing early splenic memory CD8^+^ T cells reactivation against intracellular bacteria in the mouse. *PLoS One*.

[35] Gattinoni L., Zhong X. S., Palmer D. C. (2009). Wnt signaling arrests effector T cell differentiation and generates CD8+ memory stem cells. *Nature Medicine*.

[36] Goulder P. J. R., Brander C., Tang Y. (2001). Evolution and transmission of stable CTL escape mutations in HIV infection. *Nature*.

[37] Goulder P. J. R., Phillips R. E., Colbert R. A. (1997). Late escape from an immunodominant cytotoxic T-lymphocyte response associated with progression to AIDS. *Nature Medicine*.

[38] Troyer R. M., McNevin J., Liu Y. (2009). Variable fitness impact of HIV-1 escape mutations to cytotoxic T lymphocyte (CTL) response. *PLoS Pathogens*.

[39] Liu M. K. P., Hawkins N., Ritchie A. J. (2012). Vertical T cell immunodominance and epitope entropy determine HIV-1 escape. *Journal of Clinical Investigation*.

