# Supplementary material for: IL-21 Expands HIV-1-Specific CD8+ T Memory Stem Cells to Suppress HIV-1 Replication In Vitro
Source: J Immunol Res. 2019 Apr 30;2019:1801560. doi: 10.1155/2019/1801560 (PMC6515191; doi:10.1155/2019/1801560)
Supplement: Supplementary Materials — Table I: summary of HIV-1-infected individuals. Table II: antibodies used for flow cytometry. Figure S1: gating strategy for live cells (the middle panel) and single cells (right panel). Figure S2: the Fluorescence Minus One (FMO) controls for flow cytometric analysis of HIV-1 antigen-specific CD8+ TSCMs. Figure S3: the detection of nonspecific tetramer binding. Figure S4: expression of CD19, CD14, and CD56 in CD8+ T memory stem cells (TSCMs) and peripheral blood mononuclear cells (PBMCs). Figure S5: gating strategy for the analysis of in vitro-generated CD8+ T memory stem cells (TSCMs). Figure S6: purity of CD3+CD8+CD45RO−CD45RA+CD62L+CCR7+CD95+CD122+ T cells after sorting by flow cytometry. [file 1801560.f1.pdf]

IL-21 Expands HIV-1-specific CD8$^+$ T Memory Stem Cells to Suppress HIV-1 Replication In Vitro

Kang Wu[1,2,3#], Shaoying Zhang[6#], Xu Zhang[1,2,3], Xinghua Li[5], Zhongsi Hong[5],Fei Yu[1,2,3], Bingfeng Liu[1,2,3], Ting Pan[1,2,3], Zhaofeng Huang[1,2,3], Xiao-ping Tang[4], Weiping Cai[4], Jinyu Xia[5], Xuefeng Li[1,2,3], Hui Zhang[1,2,3], Yiwen Zhang[1,2,3]* and Linghua Li[4]*

1. Institute of Human Virology,
2. Key Laboratory of Tropical Disease Control of Ministry of Education,
3. Guangdong Engineering Research Center for Antimicrobial Agent and Immunotechnology, Zhongshan School of Medicine, Sun Yat-Sen University, Guangzhou, China 510080
4. Department of Infectious Diseases, Guangzhou Eighth People's Hospital, Guangzhou Medical University, Guangzhou, China, 510060
5. Department of Infectious Diseases, Fifth Affiliated Hospital, Sun Yat-Sen University, Zhuhai, Guangdong, China,519000
6. National- Local Joint Engineering Research center of Biodiagnosis & Biotherapy, the Second Affiliated Hospital, Xi'an Jiaotong University, Xi’an, China. 710004

#These authors contribute equally to this work.

* Corresponding authors:

Dr. Linghua Li, Department of Infectious Diseases, Guangzhou 8th People’s Hospital Guangzhou, Guangdong, 510080, China. E-mail: llheliza@126.com

Dr. Yiwen Zhang, Institute of Human Virology, Zhongshan School of Medicine, Sun Yat-sen University, Guangzhou, 510080, China. E-mail: zhangyw57@mail.sysu.edu.cn

Data Availability Statement: The clinical samples data used to support the findings of this study are included within the article.

Supplemental Materials

Table Ⅰ HIV-1-infected individuals

Number	Gender	Age	cART	HIV-RNA	CD4 counts (cells/mm^3)
1	Male	44	TDF+3TC+EFV	＜20	150
2	Male	36	TDF+3TC+EFV	＜20	159
3	Male	26	TDF+3TC+EFV	＜20	272
4	Male	33	AZT/3TC+EFV	＜20	85
5	Female	45	AZT/3TC+EFV	＜20	199
6	Male	34	AZT/3TC+EFV	＜20	499
7	Male	48	TDF+3TC+EFV	＜20	196
8	Male	59	TDF+3TC+EFV	＜20	345
9	Male	44	AZT/3TC+EFV	＜20	467
10	Male	45	TDF+3TC+EFV	＜20	433
11	Female	37	TDF+3TC+EFV	＜20	155
12	Female	28	AZT/3TC+EFV	＜20	41
13	Male	28	TDF+3TC+EFV	＜20	432
14	Male	44	TDF+3TC+EFV	＜20	232
15	Male	33	TDF+3TC+EFV	＜20	328
16	Male	42	TDF+3TC+EFV	＜20	272
17	Male	33	AZT/3TC+EFV	＜20	290
18	Female	55	AZT/3TC+EFV	＜20	176
19	Male	36	AZT/3TC+EFV	＜20	448
20	Male	43	TDF+3TC+EFV	＜20	173

TDF: Tenofovir disoproxil fumarate

AZT: Azidothymidine

EFV: Efavirenz

3TC : Lamivudine

Table Ⅱ Antibodies in flow cytometry

Target	Clone	Labeling
**CD3**	OKT3	PE
**CD4**	OKT4	FITC
**CD8**	RPA-T8	Pacific Blue
**CD45RA**	HI100	PE/Dazzle 594
**CD45RO**	UCHL1	Percp/Cy5.5
**CD62L**	DREG-56	PE-Cy7
**CCR7**	G043H7	Alexa Fluor ® 700
**CD95**	DX2	FITC
**CD122**	TU27	APC

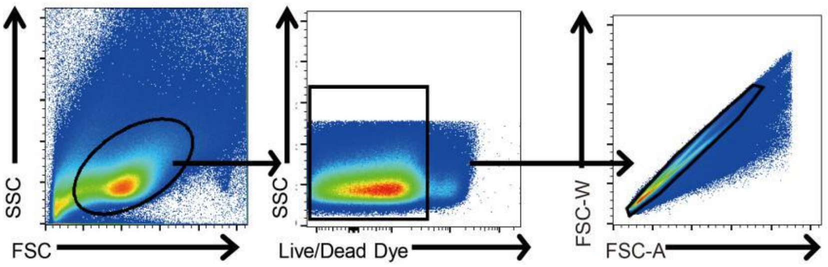

Fig S1. The gating strategy for live cells (the middile panel) and single cells (right panel).

The forward scatter (FSC) and side scatter (SSC) show the properties of the cell size and density of PBMCs in HIV-1 individuals. Dot plots represent the strategy of flow cytometric analysis of single cells and live cells. The Live/Dead Dye graph represents the live cells (negative subset) and dead cells (positive subset).

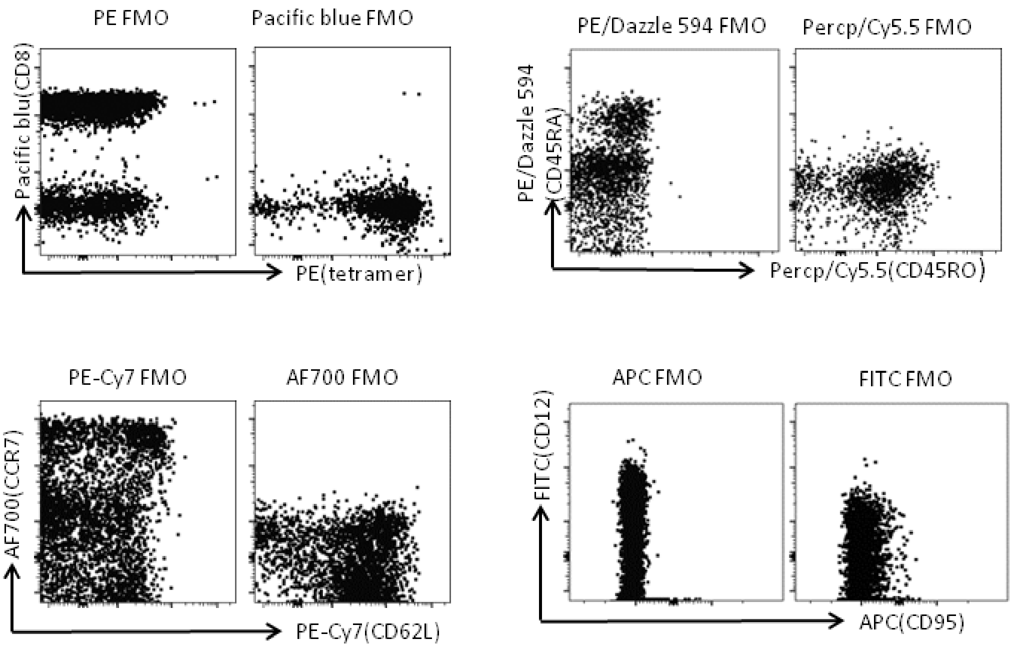

Figure S2. The Fluorescence Minus One Controls (FMO) for flow cytometric analysis of HIV-1 antigen specific CD8$^+$ T$_{SCM}$s.

The dot plots showed the FMO for corresponding parameters (fluorescence stainings and corresponding molecules) in flow cytometric analysis of HIV-1 antigen specific CD8$^+$ T$_{SCM}$s.

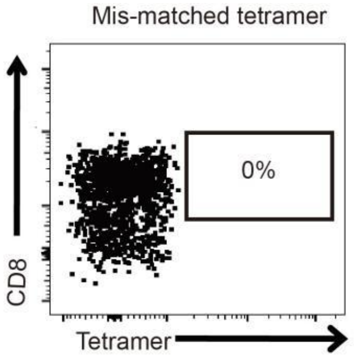

Fig S3. The detection of the non-specific binding of tetramer.

The mis-matched tetramer with nonsense HLA subtype (HLA-A2-restricted RYLRDQQLL) as a non-specific binding control tetramer to test the non-specific binding of tetramers in peripheral blood mononuclear cells (PBMCs) from human immunodeficiency virus type 1 **(**HIV-1)-infected individuals. Dot plot showed the gating strategy to detect the non-antigen-specific binding of tetramers in PBMCs from

HIV-1-infected individuals. The number in the graph represents the percentage of $CD8^+$ $tetramer^+$ cells in HIV-1 PBMCs. The data are representative from five independent experiments (n=6).

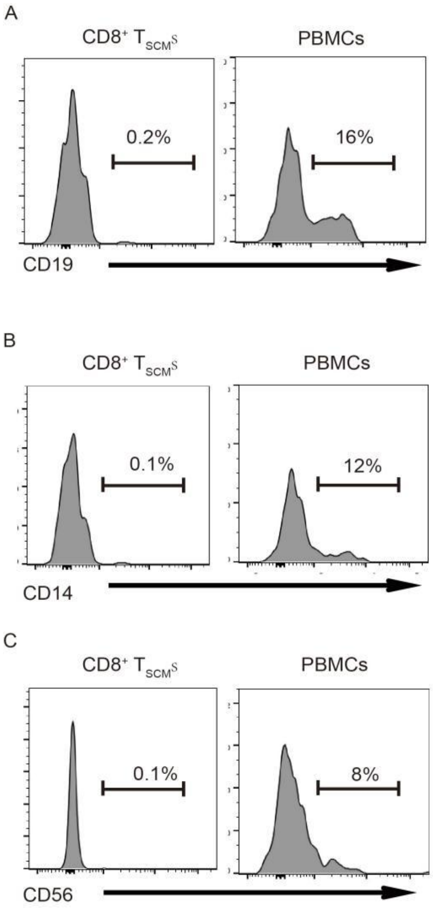

Fig S4. The expressions of CD19, CD14 and CD56 in $CD8^+$ T_{SCM}s and PBMCs.

The expressions of CD19, CD14 and CD56 molecules in $CD8^+$ T_{SCM}s or PBMCs of HIV-1 infecred individuals were tested by flow cytometry. Dot plots show the expression levels of corresponding molecules in $CD8^+$ T_{SCM}s and PBMCs. Data are representative for three independent experiments (n=3).

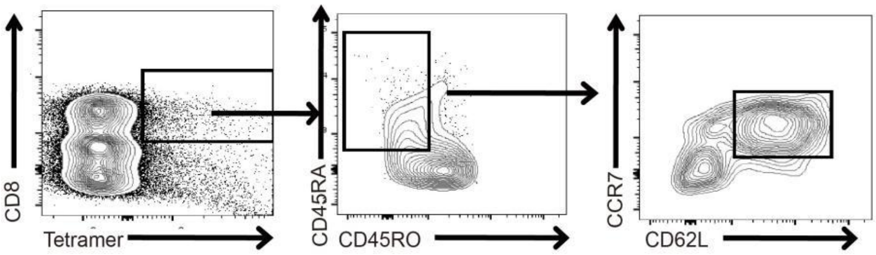

Fig S5. Gating strategy for the analysis of the in-vitro generated $CD8^+$ T_{SCM}s.

The gating of CD95^{+}CD122^{+} T cells in tetramer^{+}CD8^{+}CD45RO^{-}CD45RA^{+} CD62L^{+}CCR7^{+} cells compartment was performed. Dot plots represent the strategy of flow cytometric analysis of the in-vitro generated CD8^{+} T_{SCM}s.

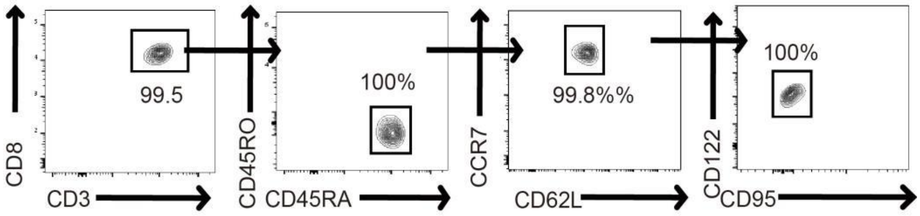

Fig S6. The purity of CD3^{+}CD8^{+}CD45RO^{-}CD45RA^{+}CD62L^{+}CCR7^{+}CD95^{+} CD122^{+} T cells after sorted by flow cytometry.

The CD3^{+}CD8^{+}CD45RO^{-}CD45RA^{+}CD62L^{+}CCR7^{+}CD95^{+}CD122^{+} T cells were sorted by flow cytometry. Numbers in dot plots show that the percentage of CD3^{+}CD8^{+}CD45RO^{-}CD45RA^{+}CD62L^{+}CCR7^{+}CD95^{+}CD122^{+} cells.
